# Network and Evolutionary Analysis of Human Epigenetic Regulators to Unravel Disease Associations

**DOI:** 10.3390/genes11121457

**Published:** 2020-12-04

**Authors:** Shinji Ohsawa, Toshiaki Umemura, Tomoyoshi Terada, Yoshinori Muto

**Affiliations:** 1 United Graduate School of Drug Discovery and Medical Information Sciences, Gifu University, 1-1, Yanagido, Gifu 501-1193, Japan; v3502001@edu.gifu-u.ac.jp (S.O.); tterada@gifu-u.ac.jp (T.T.); 2 Department of Nursing, Ogaki Women’s College, 1-109, Nishinokawa-cho, Ogaki 503-8554, Japan; 3 Graduate School of Medicine and Pharmaceutical Sciences, University of Toyama, 2630, Sugitani, Toyama 930-0194, Japan; tumemura@med.u-toyama.ac.jp; 4 Department of Functional Bioscience, Gifu University School of Medicine, 1-1, Yanagido, Gifu 501-1193, Japan

**Keywords:** protein–protein interaction, cancer disease, developmental disease, co-expression module, evolutionary rate, positive selection, antagonistic pleiotropy

## Abstract

We carried out a system-level analysis of epigenetic regulators (ERs) and detailed the protein–protein interaction (PPI) network characteristics of disease-associated ERs. We found that most diseases associated with ERs can be clustered into two large groups, cancer diseases and developmental diseases. ER genes formed a highly interconnected PPI subnetwork, indicating a high tendency to interact and agglomerate with one another. We used the disease module detection (DIAMOnD) algorithm to expand the PPI subnetworks into a comprehensive cancer disease ER network (CDEN) and developmental disease ER network (DDEN). Using the transcriptome from early mouse developmental stages, we identified the gene co-expression modules significantly enriched for the CDEN and DDEN gene sets, which indicated the stage-dependent roles of ER-related disease genes during early embryonic development. The evolutionary rate and phylogenetic age distribution analysis indicated that the evolution of CDEN and DDEN genes was mostly constrained, and these genes exhibited older evolutionary age. Our analysis of human polymorphism data revealed that genes belonging to DDEN and Seed-DDEN were more likely to show signs of recent positive selection in human history. This finding suggests a potential association between positive selection of ERs and risk of developmental diseases through the mechanism of antagonistic pleiotropy.

## 1. Introduction

The term epigenetics refers to heritable traits that are not attributable to changes in the DNA sequence, and this concept has attracted much attention as a potential mechanism underlying the regulation of various cellular processes [1]. Several epigenetic regulatory mechanisms, such as DNA methylation, histone post-translational modifications, chromatin remodeling, histone protein variants, and long noncoding RNA, have been shown to control DNA and chromatin structures [2,3], which in turn dictate specific gene expression programs and regulate cellular and organismal functions [4]. It is important to note that such epigenetic changes are plastic, which enables cellular reprogramming and response to the environment. This dynamic nature of epigenetics means that epigenetic changes may not only alter the cellular differentiation and development of an organism, but also, if controlled inappropriately, induce various severe disease states [5,6]. Therefore, there is a great need for information about epigenetic regulations and their components in order to fully understand human disorders and their treatment.

Over the past decade, extensive researches have been directed towards understanding epigenetic regulatory mechanisms, including various individual components in the regulatory machinery. These components, referred to as epigenetic regulators (ERs) [7], are usually classified into three categories according to their regulatory roles in epigenetics: DNA modifiers, histone modifiers, and chromatin remodelers [8,9]. While DNA modifiers methylate and demethylate the cytosine bases of DNA molecules, histone modifiers covalently modify amino acid residues of histone molecules. In contrast, chromatin remodelers are a different type of ER that decouple the association between nucleosomes and DNA, and replace or move nucleosomes in the chromatin [3,8,10]. Mutation and deregulation of these ERs are observed in many complex human diseases, including cancers, neurodegenerative diseases, and developmental disorders [11,12,13]. Recently, many ER genes have been demonstrated to be very intolerant to loss-of-function variation, even when compared to the dosage-sensitive transcription factors, underscoring the important roles of ERs in various diseases [14]. By integrating numerous studies and curated databases, more than several hundred ERs and their sources have been compiled [8,9]. However, very few studies have systematically investigated the protein–protein interaction (PPI) networks as well as the evolutionary properties of ERs that may link to human disease, and to our knowledge there are no prior studies looking at co-expression modules associated with ERs in the developmental transcriptome.

In this study we examined associations between epigenetic regulators and human diseases in the context of PPI networks using the DisGeNET database, which integrates information on human diseases and their associated genes [15]. We verified that diseases associated with ERs are mainly clustered into two large groups, cancer diseases and developmental diseases. ERs associated with these disease clusters formed highly interconnected PPI networks that were characterized by unique gene ontology (GO) sets. We found that whereas disease-associated ERs showed a general pattern of evolutionary conservation, they were also enriched for signatures of positive selection, as inferred from polymorphism data. In addition, ER-associated co-expression modules identified in the early developmental stages of mice exhibited a distinct pattern of expression profiles for each disease group. These genome-wide assessments will yield new insights into the pathways and roles of ERs in human diseases, providing a useful tool and clues for further investigations of cancer and developmental diseases.

## 2. Materials and Methods

### 2.1. Collection of Epigenetic Regulators (ERs)

The data sets used to compile the genes encoding the ERs were downloaded in October 2018 from the EpiFactors database (available at http://epifactors.autosome.ru/) [9] and the Functional Atlas of Chromatin Epigenetic Regulators (FACER) (available at http://bio-bigdata.hrbmu.edu.cn/FACER/) [8]. The EpiFactors and FACER databases are manually curated databases providing information about 815 and 870 epigenetic regulators, respectively. In addition, in order to obtain as much comprehensive information on the ERs as possible, we downloaded the lists of proteins under the following three related GO terms from QuickGO (https://www.ebi.ac.uk/QuickGO/) in October 2018: “DNA modification” (GO:0006304), “Histone modification” (GO:0016570), and “Chromatin remodeling” (GO:0006338). These three data sets provide overlapping and complementary candidate epigenetic regulators. We combined the EpiFactors genes, FACER genes, and QuickGO genes to construct a comprehensive list of epigenetic regulators. Since histone and protamine genes are direct targets of ERs, we included 95 histone and protamine genes from the EpiFactors database in the final data set, which contained a total of 1001 genes (Supplementary Table S1).

### 2.2. Curation and Clustering of Diseases Enriched for the ER Gene Set

The list of diseases enriched for ER genes was retrieved from the DisGeNET database by using ToppFun of the ToppGene Suite (available at https://toppgene.cchmc.org/) [16]. DisGeNET contains gene-disease and variant-disease associations from different sources, including curated and text mining data [15]. For our analysis, gene-disease associations of curated data with more than 5 co-occurrences of ERs and a false discovery rate (FDR) greater than 0.05 were used as enriched diseases for ERs. Hypergeometric distribution with Benjamini–Hochberg correction was used as the method for determining statistical significance (Supplementary Table S2).

The disease enrichment results were visualized using the EnrichmentMap (v3.1.0) plugin [17] for the program Cytoscape (v3.7.1, https://cytoscape.org/) [18]. The diseases enriched for ER genes were mapped as a network of diseases where the nodes represent statistically significant diseases and the edges represent the degree of disease gene set similarity; Jaccard metrics with a default cutoff of 0.25 were employed. Diseases that shared associated ER genes were clustered by AutoAnnotate software (v1.2) according to the Markov cluster (MCL) algorithm and were automatically annotated using the WordCloud algorithm [19].

### 2.3. Construction of a Protein–Protein Interaction (PPI) Network

A human PPI network was constructed using binary protein interaction datasets essentially as described previously [20]. Briefly, protein interaction data was downloaded from the iRefIndex database (https://irefindex.vib.be/wiki/index.php/iRefIndex) as an initial data set [21]. To increase the confidence and the completeness of the PPI network, we integrated PPIs from the large scale BioPlex 2.0 interaction data set [22] obtained by high-throughput affinity purification mass spectrometry. In addition, we incorporated interaction data other than direct physical bindings from a previously published paper [23] into our data set. The final data set was filtered to remove interactions from non-human sources and the redundant and self-interacting pairs were excluded. A full list of the human PPI network data is provided as Supplementary Table S6.

### 2.4. PPI Subnetworks among Proteins Encoded by Disease-Associated ER Genes

We used the 100 top-ranking ER genes with respect to the number of diseases shared in the cancer disease and developmental disease clusters separately as seed genes and mapped them to the human PPI network as described above, and then extracted the maximal connected component as the protein interaction subnetwork. To enhance our understanding of the local neighborhood of member ERs in the subnetworks, we applied the disease module detection (DIAMOnD) method, which iteratively expands the seed gene neighborhood by adding proteins with a significant number of connections to the seed gene pool [24]. Each cancer disease and developmental disease network was expanded separately using the settings recommended for the DIAMOnD algorithm, including running 100 iterations. Full lists of the cancer disease ER network data and the developmental disease ER network data are provided as Supplementary Table S7a,b.

### 2.5. PPI Subnetworks Simulation

The connectivity of the PPI subnetworks of ERs was quantified by the size of the largest connected component (LCC) and shortest distance (SD) between proteins [23]. We calculated the LCC, SD, and significance of each subnetwork using the Python scripts as described previously [20]. To assess the significance of LCC and SD, expected null distributions were calculated by randomizing sets of proteins of equal seed list size. A total of 1000 random gene sets were generated. The z-score was calculated to estimate whether the two network parameters of disease-associated ER genes were significantly different from the 1000 simulated networks.

### 2.6. Tissue-Specific Gene Expression Analysis

The tissue specificity of gene expression among ERs and disease gene clusters was examined using a published gene expression data set generated with tissue samples of 122 human individuals representing 32 different tissues [25]. Gene expressions for individual tissues were represented by using FPKM (fragments per kilobase of exon model per million mapped reads). According to Uhlen et al., genes were classified into one of six categories based on their expression patterns in 32 tissues: (1) Not detected, FPKM < 1 in all tissues; (2) expressed in all tissues, detected in all 32 tissues with FPKM > 1; (3) tissue-enriched, at least a 5-fold higher FPKM level in one tissue compared to all other tissues; (4) group-enriched, a 5-fold higher average FPKM value in a group of 2–7 tissues compared to all other tissues; (5) tissue-enhanced, at least a 5-fold higher FPKM level in one tissue compared to the average value of all 32 tissues; and (6) mixed, the remaining genes were detected in 1–31 tissues with FPKM > 1 and in none of the above categories [25]. We used 19,986 human protein-coding genes from the GENCODE annotation (version 30) as the background in the present analysis [26].

### 2.7. Weighted Gene Co-Expression Network Analysis (WGCNA)

To generate a gene co-expression network, we used previously published RNA-seq data from a genome-wide expression analysis over the 2-cell stage to E (embryonic day) 18.5 of mouse embryogenesis [27]. We obtained processed (normalized) RNA-seq data from Hu et al. [27], and log-converted the FPKM values using R software. The WGCNA package implemented in R [28,29] was used to construct a signed weighted gene co-expression network based on the expression values of 39 RNA-seq data sets. Non-informative genes were excluded based on the standard deviation from the mean of the genes, retaining 50% of the total genes with highly variable expression values (*N* = 19,187 remaining genes). Network construction was carried out using the blockwiseModules function in the WGCNA package. A power of 12, at which the scale-free topology fit index curve flattens at about 0.9, was interpreted as a soft threshold for the adjacency matrix. The minimum module size was set to 30 genes and the minimum height for merging modules was set to 0.25. In total, 21 modules were identified (Supplementary Table S8). Each module was summarized by a single representative expression profile called a module eigengene. The module eigengene is an integrative representation of the gene co-expression relationships in each network module [30]. To assess the associations between modules and developmental stages, we quantified eigengenes at various stages of development. To identify modules enriched for ER-related disease gene sets, we used one-sided Fisher’s exact test according to the R function fisher.test.

### 2.8. Estimation of Evolutionary Rates and Gene Evolutionary Origin

The dN/dS ratios of human genes were calculated by dividing the nonsynonymous substitution rate (dN) by the synonymous substitution rate (dS). Human-mouse pairwise dN and dS were obtained from the Ensembl BioMart interface [31]. Only the 1:1 orthologs were selected. To calculate the dN/dS ratio, only the orthologs with nonzero pairwise dS were kept. Tajima’s *D* calculated over the exonic sequences of each gene was downloaded from the supplementary data in a previous work [32]. Estimates of the evolutionary age of human genes were made with ProteinHistorian [33]. We used the Wagner parsimony algorithm on the PPODv4_OrthoMCL_families database according to the default settings of the ProteinHistorian server (https://lighthouse.ucsf.edu/ProteinHistorian/). The evolutionary ages of genes were also retrieved from previously published phylostratigraphy analysis [34,35], which assigned human genes onto a phylogenetic tree of 16 phylostrata, spanning genes found across cellular organisms, in other words, phylostratum 1, to those specific to humans, phylostratum 16.

### 2.9. Analysis of Positive Selection Using Divergence and Polymorphism Data

To obtain evidence of positive selection inferred from divergence data, we retrieved the results of site model and branch-site model tests from previously published PAML analysis [36]. The analysis used a set of primate sequences of 15,037 ortholog clusters to infer events of positive selection that have occurred during the evolution of primates. We used the results of site model tests (model M7/M8), which allow ω to vary across sites, but not across branches [37]. We also used the results of branch-site model tests (model A1/A), which allow ω to vary across both sites and branches leading to human lineage. For each analysis, a likelihood ratio test was performed and *p*-values were computed. The false discovery rate was estimated with *q*-values.

To identify signatures of recent positive selection inferred from human polymorphism data, we downloaded the lists of genomic regions showing signatures of positive selection based on population genetic statistics from the PopHumanScan database [38]. The PopHumanScan database integrated eight different population genetic statistics for 22 non-admixed human populations of Phase III of the 1000 Genomes Project to detect selective sweeps at different historical ages. We used only positively selected regions overlapped with protein-coding genes. We also considered positive-selection scores derived from the hierarchical boosting (HB) algorithm developed by Pybus et al. (2015) [39]. The HB algorithm is a machine-learning classification framework that combines the functionality of several selection tests to measure whether the genomic region is under the positive selection expected under hard selective sweeps [39]. The HB scores calculated for the three populations of the 1000 Genomes Project Phase 1—Utah residents with Northern and Western European Ancestry (CEU); Han Chinese in Beijing, China (CHB); and Yoruba from Ibadan, Nigeria (YRI)—were downloaded from the 1000 Genomes Selection Browser 1.0 [40]. We assigned the HB scores to the protein-coding genes in GENCODE annotation (version 30) based on their genomic coordinates. The maximum value of all the regions overlapping a gene coordinate ±10 kb was used as a measure of HB score for that gene. In the present study, we considered two different positive-selection scenarios: complete selection (loci where a selected allele reached fixation) and incomplete selection (loci where a selected allele has not yet reached fixation).

### 2.10. Pathway and GO Enrichment Analysis

In order to investigate gene clusters at the molecular and functional level, we performed a Reactome pathways and GO biological process (BP) terms enrichment analysis by using clusterProfiler (http://bioconductor.org/packages/release/bioc/html/clusterProfiler.html) with a strict cut-off of FDR < 0.05 [41]. We implemented the compareCluster function in the clusterProfiler package, which calculates and compares enriched functional categories of each gene cluster. Using gene ratios (the proportions of genes enriched in each category) and adjusted *p*-values, we constructed a dot plot to visualize the difference of enriched functional categories between the clusters.

## 3. Results

### 3.1. Identification of ER Genes Associated with Various Human Disorders

To investigate the properties of epigenetic regulators (ERs) associated with human disease, we first compiled a comprehensive set of ERs from two high-quality data sources: the EpiFactors database [9] and the Functional Atlas of Chromatin Epigenetic Regulators (FACER) [8]. In addition to these well-curated gene lists, annotated human proteins under the related GO terms were also retrieved from the GO database (see Materials and Methods). After removing duplicated and multiple genes, a total of 1001 human ER genes remained and were used as the foundation for our study (Supplementary Table S1). Overall, the final ER genes were grouped into four functional categories: 205 chromatin remodeling (20.5%), 69 DNA modification (6.9%), 632 histone modification (63.1%), and 95 histone and protamine (9.5%) genes (Supplementary Table S1).

**Figure 1:**
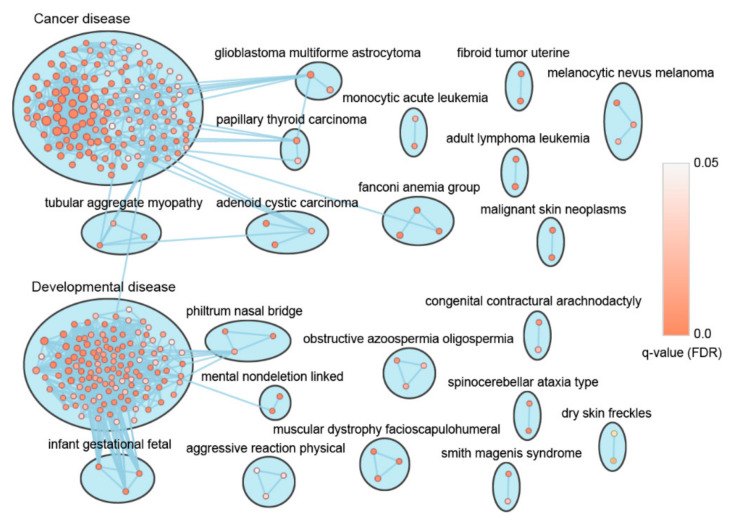
Disease-disease association network of diseases enriched for epigenetic regulators (ERs). A disease network formed by 384 diseases (nodes) and 1688 edges was clustered using the Markov cluster (MCL) algorithm with Jaccard metrics. The node size is proportional to the gene number of the node. The node color denotes enrichment significance—the deeper the color, the higher the enrichment significance (FDR *q* < 0.05). Cytoscape and EnrichmentMap were used for visualization of the results.

We next mapped all the ER genes to the disease in the DisGeNET curated data set [15] using the ToppGene search program [16]. A total of 457 diseases were enriched with ER genes at a *q*-value greater than 0.05, and the number of genes associated with a disease ranged from 2 to as high as 199 for leukemia, with 684 ER genes in all (Supplementary Table S2). To construct a disease network, we used 384 diseases enriched for more than 5 co-occurring ER genes. As shown in Figure 1, the network was composed of 384 nodes representing diseases and 1688 interconnecting edges representing their gene set similarity (see Materials and Methods). Clustering the disease network by gene list similarity resulted in 22 clusters, including two giant clusters with 150 and 130 diseases each and several smaller ones with 2 or 3 diseases each (Figure 1). It is noteworthy that the largest cluster was mostly composed of cancer diseases, whereas the second largest cluster contained various developmental diseases (Supplementary Table S3). Here we characterize the two large clusters of cancer diseases and developmental diseases for further analysis.

### 3.2. Functional Characterization of the Cancer Disease and Developmental Disease Clusters

**Figure 2:**
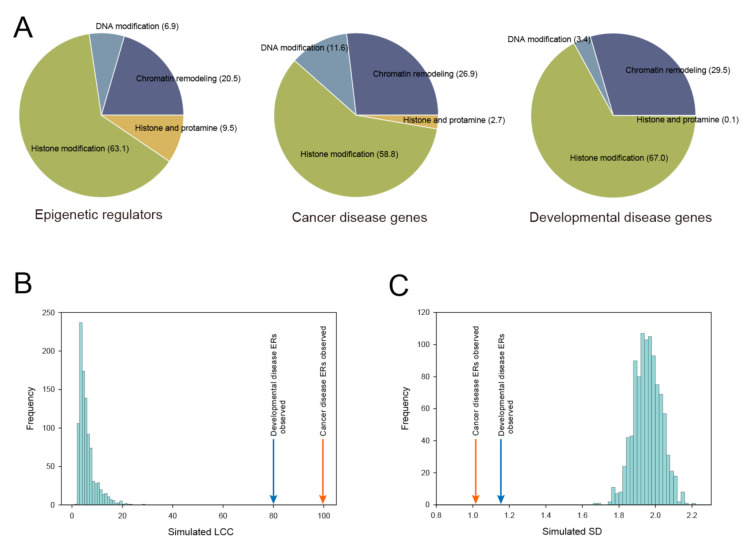
The functional features and network properties of the cancer disease and developmental disease clusters. (**A**) Distribution of ERs in different categories. Charts show the proportion of ERs observed in each category. (**B**) Largest connected component (LCC) size of the cancer disease seed genes (orange arrow) and developmental disease seed genes (blue arrow) in comparison to the expected, random distribution (histogram). The simulation results of 1000 random networks are shown in the histograms. (**C**) Shortest distance (SD) measure of the cancer disease seed genes (orange arrow) and developmental disease seed genes (blue arrow) in comparison to the expected, random distribution (histogram). The simulation results of 1000 random networks are shown in the histograms.

In order to interpret the functional cross-links between diseases and ER genes, we compared the cancer disease and developmental disease clusters by extracting associated ER genes. Gene category analysis of the ERs in the disease clusters demonstrated that the majority of disease-associated ERs were histone modifiers as is the case with all the human ER genes (Figure 2A). In contrast, the percentage of DNA modifiers was increased in the cancer disease cluster (*p* < 0.001, Chi-square test), but decreased in the developmental disease cluster (*p* < 0.001, Chi-square test) (Figure 2A). Histone and protamines were depleted in both the cancer disease cluster and the developmental disease cluster (*p* < 0.001, Chi-square test) (Figure 2A). Since ER genes that are highly shared among individual diseases in the cluster might play essential and typical functions in disease development, we focused on the 100 genes that were the most highly shared among diseases in the cancer disease cluster and the 100 that were most highly shared among diseases in the developmental disease cluster (Supplementary Table S4). We used these 100 top-ranking genes as seed genes for further PPI analysis. To identify pathways associated with the seed genes, we performed a Reactome pathway analysis using clusterProfiler. The two most significantly overrepresented pathways common to the lists of both seed genes were covalent chromatin modification and histone modification (Supplementary Figure S1B). In addition, specific pathways for the cancer disease seed genes, such as the pathways for the regulation of the DNA metabolic process and DNA modification, were also evident. These annotation results for the seed genes were consistent with the significant overlap of the two gene sets (Supplementary Figure S1A).

### 3.3. Construction of the Cancer Disease ER Network and Developmental Disease ER Network

We investigated the PPI subnetworks among proteins encoded by cancer disease-related ER genes and developmental disease-related ER genes by using the human molecular interaction network. As a molecular interaction data source, we compiled the human PPI network from several data sets containing physical binary interactions among molecular components (see Materials and Methods). In total, a human PPI network containing 22,616 nodes and 515,015 edges was constructed. We mapped the 100 top-ranking ER genes as seed genes to the human PPI network and measured the size of the largest connected component (LCC) to quantify the degree to which the proteins tended to agglomerate [23]. To assess whether the proteins encoded by ER genes interacted significantly and agglomerated with each other, we also compared the observed LCC size with randomly permutated LCC sizes by computing z-scores. In the present analysis, LCCs of the cancer disease-related ERs and the developmental disease-related ERs were significantly larger than expected by random chance (cancer disease ERs: LCC = 99, z-score = 26.9; developmental disease ERs: LCC = 80, z-score = 21.9) (Figure 2B). In addition to the size of LCCs, we also compared the shortest distance (SD) of networks encoded by disease ER genes and random genes. We found that the SD values of cancer disease ERs and developmental disease ERs were shifted towards 1.0 and the observed values were significantly smaller compared with the random genes (cancer disease ERs: SD = 1.01, z-score = −12.7; developmental disease ERs: SD = 1.16, z-score = −11.1) (Figure 2C). Taken together, the significant network measurements demonstrate that protein products of cancer disease-related ER genes and developmental disease-related ER genes exhibit a high tendency to interact and agglomerate. Interestingly, this tendency to mutually agglomerate was more significant in cancer disease ERs than in developmental disease ERs, suggesting that cancer disease ERs more coherently contribute to disease risk through their mutual interactions.

**Figure 3:**
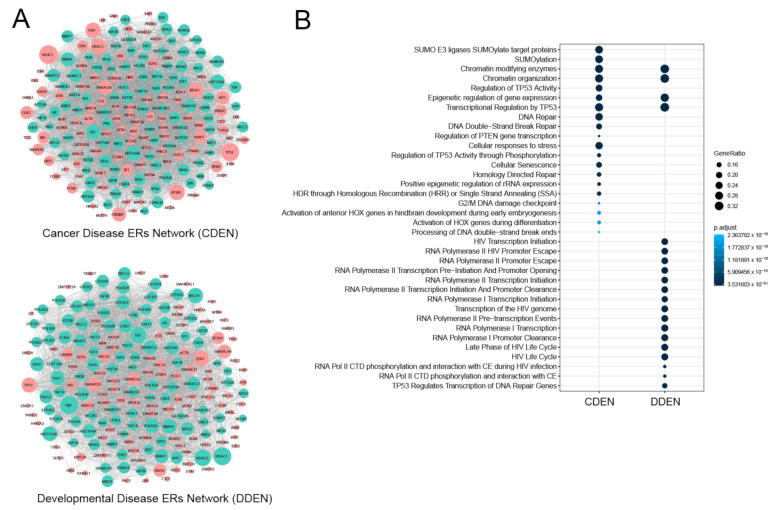
Cancer disease ER network (CDEN) and developmental disease ER network (DDEN). (**A**) PPI networks of CDEN and DDEN. Red nodes represent disease seed ER genes and green nodes represent the neighborhoods of the seed gene identified by the DIAMOnD algorithm. The node size is proportional to its degree. (**B**) Pathway analysis of the CDEN and DDEN data sets. Reactome pathway terms were determined for each gene set using the compareCluster function in the R package clusterProfiler. The most over-represented Reactome terms are illustrated as dot plots, with the gene ratio denoted by size and the significance denoted by color. *p*-values were adjusted by the Benjamini–Hochberg method.

We next identified the neighborhoods of the seed gene subnetworks and expanded them into a more comprehensive cancer disease ER network and developmental disease ER network using the disease module detection (DIAMOnD) method [24]. The DIAMOnD program identifies the topological neighborhood of the seed proteins based on the significance of connections to the seed proteins and iteratively expands the seed by adding neighbor proteins. This approach has been successfully used to study various disease interactomes [42,43]. We found that after performing 100 iterations of the DIAMOnD algorithm, the final networks consisted of 199 protein nodes and 4125 interactions for cancer disease ERs and 195 protein nodes and 3424 interactions for developmental disease ERs (Figure 3A). We call these the cancer disease ER network (CDEN) and developmental disease ER network (DDEN). To elucidate the underlying biological function, we performed Reactome pathway analysis of the proteins within CDEN and DDEN. As shown in Figure 3B, we confirmed four significantly enriched pathways common to both the CDEN and the DDEN, which were the pathways for chromatin-modifying enzymes, chromatin organization, epigenetic regulation of gene expression and transcriptional regulation by TP53, as expected from the seed gene lists. In contrast, while the pathways such as SUMOylation, DNA repair, and cellular responses to stress were specifically enriched with CDEN genes, the pathways such as HIV transcription initiation, RNA polymerase II promoter escape, and RNA polymerase II transcription initiation were specifically enriched with DDEN genes (Figure 3B). Thus, although epigenetic regulatory pathways were common to both disease subnetworks, some pathways were clearly distinct between the CDEN and the DDEN, reflecting specific gene members in those networks. Full lists of the significantly enriched Reactome pathways and their associated proteins are provided as Supplementary Table S5.

### 3.4. Tissue-Dependent Expression Levels of the CDEN and the DDEN Gene Sets

Specific epigenetic regulations are performed in individual tissues, and tissue-dependent expressions of ERs might be intimately associated with disease development [8,44]. To understand the expression levels of the CDEN and the DDEN genes in various tissues, we analyzed their expression levels across 32 different tissues [25]. Overall, we found that the ER genes were more highly expressed than the background genes in each of the 32 tissues (Supplementary Figure S2A). Strikingly, the CDEN and the DDEN gene set had much higher expression levels in all 32 tissues compared with the ERs and background genes (Supplementary Figure S2A). These high expression levels suggest that the CDEN and DDEN genes are transcriptionally active in all 32 tissues and may play critical roles in different human tissues. Gene category analysis based on expression patterns in the 32 tissues showed that a majority (>80%) of the CDEN and DDEN genes were expressed in all tissues, with only a fraction of genes expressed in a tissue- or group-enriched manner (Supplementary Figure S2B). The lack of tissue specificity for many of the CDEN and DDEN genes indicates that the corresponding proteins are involved in basic cellular functions such as growth regulation, cell differentiation and cell cycle control. Thus, the results revealed almost no overt tissue specificity of the CDEN and the DDEN genes, suggesting these genes may play various roles during cancer and developmental disease induction across a wide range of tissue types.

### 3.5. Identification of Co-Expression Network Modules Associated with the CDEN and the DDEN

**Figure 4:**
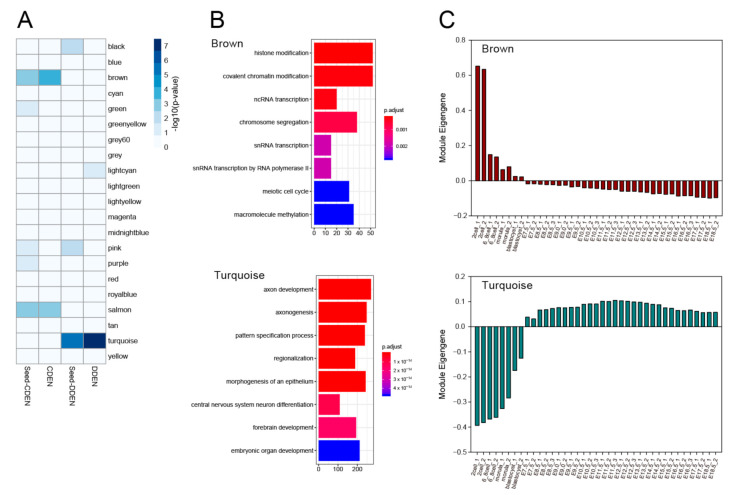
Co-expression network modules associated with the CDEN and the DDEN. (**A**) Module-level enrichment for gene sets from CDEN, its seed genes, and DDEN and its seed genes. The blue colors in each cell indicate the *p*-value for significance of overlap using Fisher’s exact test (−log_10_(*p*)). (**B**) Functional enrichment analysis results of the brown and the turquoise module genes. The gene ontology terms enriched in biological processes (BPs) were determined for each gene set using the R package clusterProfiler. The top eight BP terms are illustrated as bar plots, with the gene count denoted by the length of the bar and significance denoted by color. *p*-values were adjusted by the Benjamini–Hochberg method. (**C**) Eigengene expression patterns for the brown and turquoise modules during different stages of mouse embryonic development.

To elucidate specific features of the CDEN and the DDEN genes, we considered that it would be quite useful to identify a group of co-regulated genes, or modules. Using the transcriptome from early mouse developmental stages, we conducted a weighted gene co-expression network analysis [28,29] that identified the modules of co-expressed genes at various stages of development. This analysis identified 21 co-expression modules (Supplementary Figure S3), which were tested for enrichment of the CDEN and the DDEN gene set as well as the seed gene set. Of the 21 network modules, the brown and salmon modules were significantly enriched for both CDEN and its seed genes, while the turquoise module was significantly enriched for both DDEN and its seed genes (Figure 4A). We next investigated the identified top co-expression modules, brown for CDEN and turquoise for DDEN, as representative modules that recapitulated molecular processes related to embryonic development. GO biological process analysis revealed that the brown module was significantly enriched for genes involved in histone modification, covalent chromatin modification, ncRNA transcription, and chromosome segregation (Figure 4B). In contrast, the turquoise module was significantly enriched for genes involved in axon development, axonogenesis, pattern specification processes, regionalization, and morphogenesis of the epithelium (Figure 4B). The expression pattern of each module is summarized by a module eigengene, which is analogous to the first principal component of the module expression data [30]. Figure 4C shows the temporal expression profiles of the module eigengenes for the brown and turquoise modules for each mouse developmental stage. Consistent with the distinct GO biological process, the two modules showed different expression patterns from embryos of the 2-cell stage to E18.5, especially before and after the early blastocyst stages of development. Expression of the brown module eigengene peaked from the 2-cell to blastocyst stage, and thereafter remained relatively repressed. On the other hand, the turquoise module showed decreased expression from the 2-cell to blastocyst stage, and thereafter showed increased expression. This type of opposite pattern of eigengene expression suggests that the brown and the turquoise module genes are differentially regulated after the early developmental stages and play distinct roles during disease development.

### 3.6. Selective Pressure and Evolutionary Ages of the CDEN and DDEN Gene Sets

**Figure 5:**
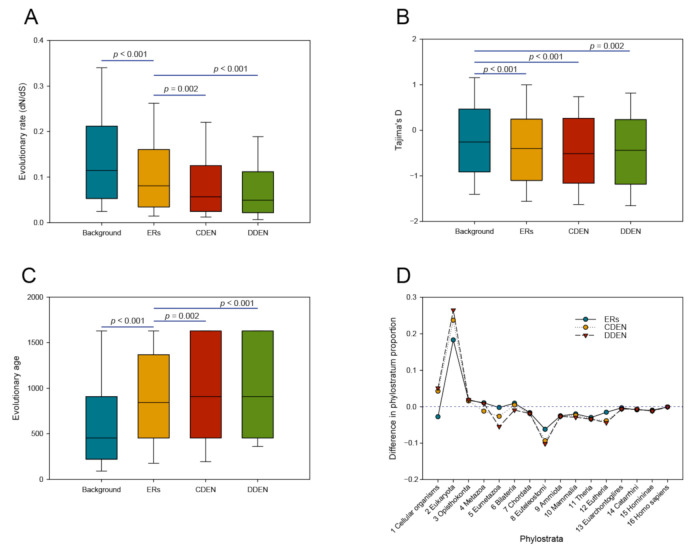
Evolutionary properties of the CDEN, DDEN, and ER genes. (**A**) Box plot of the evolutionary rates (dN/dS ratio) for the CDEN, DDEN, ER, and background genes. (**B**) Box plot of the Tajima’s *D*-values for the CDEN, DDEN, ER, and background genes. (**C**) Box plot of the evolutionary ages for the CDEN, DDEN, ER, and background genes. The evolutionary ages of human genes were estimated with ProteinHistorian. (**D**) Difference in the phylostratum proportions of the CDEN, DDEN, and ER vs. background genes across 16 phylostrata. *p* values in (**A**–**C**) were calculated by Mann–Whitney U test.

In order to gain further insight into the evolutionary features of the CDEN and DDEN gene sets, we calculated the evolutionary rate dN/dS for each of the member genes of the gene sets. We also retrieved the Tajima’s D values of the member genes from the previous work [32]. Figure 5A shows that the ER, CDEN, and DDEN gene sets have lower evolutionary rates than background genes. Moreover, the median dN/dS values of the CDEN and DDEN gene sets were significantly lower than those of the ER genes (Figure 5A; *p* < 0.005, Mann–Whitney U test), suggesting that the CDEN and DDEN genes tend to undergo strong purifying selection. The Tajima’s *D* neutrality statistic tends to detect more recent evolutionary processes, such as those impacting human populations [45]. As can be seen in Figure 5B, we observed lower negative Tajima’s *D*-values for the CDEN, DDEN, and ER gene sets compared with the background values (*p* < 0.005, Mann–Whitney U test). These negative Tajima’s *D*-values indicate an excess of low-frequency polymorphisms relative to expectation and support the idea that purifying selection occurred in human populations.

The evolutionary history of a gene often reflects its functional significance and properties. In our present analyses, we estimated the evolutionary age by referring to the approximate date that the gene originated, using a phylogenetic approach implemented by the ProteinHistorian server [33]. Figure 5C shows that the average evolutionary ages of the ER, CDEN, and DDEN genes were significantly older than those of the background genes (*p* < 0.005, Mann–Whitney U test). In particular, the evolutionary ages of the CDEN and DDEN genes were even older than those of the ER genes (*p* < 0.005, Mann–Whitney U test). We next classified genes by their evolutionary age using phylostratigraphy [34], resulting in 16 phylogenetic groups (phylostrata). We calculated the fraction of genes in each phylostratum and assessed the differences in the phylostratum proportions of ER, CDEN, and DDEN vs. background genes. As shown in Figure 5D, we found a high enrichment of ER, CDEN, and DDEN genes in the earlier phylostratum 2 (Eukaryota), with the DDEN genes being the most enriched. Collectively, these observations suggest that the CDEN and DDEN genes as well as the ER genes tend to have ancient histories, with a likely origination in unicellular eukaryotes based on the estimated ages.

### 3.7. Positive Selection Inferred from Divergence and Polymorphism Data

Consistent with previous results for human disease-associated genes [32,46,47], we observed that purifying selection is stronger in the CDEN and DDEN genes than the ERs and background genes (Figure 5A). However, in addition to the overall conserved nature of the disease genes, local nucleotide sites and lineages of the individual genes might be shaped by diverse evolutionary forces. In order to evaluate for signatures of different evolutionary forces acting on ERs associated with disease, we investigated different levels of positive selection by using divergence and polymorphism data.

**Figure 6:**
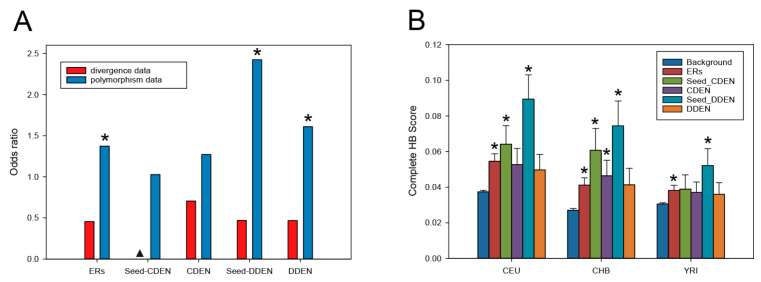
Enrichment of the signatures of positive selection in the ER genes associated with cancer and developmental diseases. (**A**) Odds ratio of positive selections versus non-positive selections for five sets of ER-related genes. We represent the odds ratios for the genes with a signal of positive selection inferred from divergence data (in red) and a signal of positive selection inferred from polymorphism data (in blue). An odds ratio greater than 1 implies that genes of the ER-related gene set are enriched with positive selections (an asterisk indicates *p* < 0.05, Fisher’s exact test). An odds ratio less than 1 implies that genes of the ER-related gene set are enriched with non-positive selections (a column marked with ▲ indicates no matched positively selected gene). (**B**) Complete hierarchical boosting (HB) scores for five sets of ER-related genes and background genes. We show the HB scores for the three populations of the 1000 Genomes Project Phase 1: Utah residents with Northern and Western European Ancestry (CEU); Han Chinese in Beijing, China (CHB); and Yoruba from Ibadan, Nigeria (YRI). An asterisk indicates *p* < 0.05 compared with background genes (Mann–Whitney U test).

We used divergence data of primate genomes [36] to infer events of positive selection with the site and branch-site models implemented in the PAML [37]. The total number of genes under positive selection, considering a *q*-value smaller than 0.2, was 249 genes for the site model and 279 genes for the branch-site model. We merged positively selected genes obtained from both models and used them as a positive-selection gene set inferred from divergence data. We then examined the enrichment of CDEN and DDEN with this gene set. Seed genes for CDEN (Seed-CDEN) and seed genes for DDEN (Seed-CDEN) were also examined in this analysis. As can be seen from the red bar in Figure 6A, odds ratios were less than one, implying that CDEN and DDEN including their seeds and ERs were extremely depleted with positively selected genes from divergence data. We next obtained the list of genes that were recently subjected to positive selection from the PopHumanScan database [38] and used it as a positive-selection gene set inferred from polymorphism data. In contrast to the results with the divergence data, all the ER-related gene groups showed a strong tendency to contain positively selected genes inferred from the polymorphism data (Figure 6A, blue bar). We observed statistically significant enrichment of the gene set of positive selection from polymorphism data in DDEN, Seed-DDEN, and ERs (Fisher’s exact test, *p* < 0.05).

To explore the strength of recent positive selection under complete (complete HB) or incomplete selective sweeps (incomplete HB) [39], we used HB scores calculated for the three populations of the 1000 Genomes Project [40]. HB scores of the complete selective sweep tend to have higher values among all the ER-related gene groups compared with those of background genes (Figure 6B). Genes of the Seed-DDEN groups exhibited the most significant HB scores in all three human populations (Mann–Whitney U test, *p* < 0.001). A similar trend of HB scores was observed under the incomplete selective sweep, but in this case the trend was less pronounced (Supplementary Figure S4).

Taken together, our observations indicate that genes encoding ERs associated with disease are more likely to show signs of recent positive selection in human history, with substantial evidence for complete selective sweeps in which favored alleles can reach fixation within the population [48]. Among the 100 genes in the most significant Seed-DDEN group, 15 genes showed evidence of positive selection by the PopHuman data (Table 1). The protein products encoded by the positively selected genes belong to the functional categories of chromatin remodeling or histone modification and are enriched in GO terms for biological processes associated with chromatin organization (*p* = 1.039 × 10^−16^) and chromosome organization (*p* = 2.594 × 10^−14^). 

## 4. Discussion

Epigenetic regulators (ERs) play crucial roles in epigenetic gene regulations, and the various molecular alterations of ERs contribute to the diverse range of human diseases [49]. The functional pathways and the affected molecular mechanisms of their action are far from being fully elaborated. In the present study, we implemented a system-level approach to link ERs to gene–disease information and the PPI network [50], and generated a subnetwork that provides unique insights into the mechanisms underlying the function of ER gene members [51]. Based on the curated gene–disease information of the DisGeNET database, we first constructed a disease network enriched with compiled ER genes. By clustering the disease network, we identified the two most prominent clusters as cancer diseases and developmental diseases. This finding supports previous observations on the involvement of epigenetic mechanisms in many cancer and developmental disorders [49,52], and it highlights the importance of ERs in the pathogenesis of these diseases. Next, to study the functional cross-links and the underlying mechanisms of disease-associated ERs, we integrated the physical interactions among proteins encoded by each of the cancer disease ER genes and developmental disease ER genes with the use of the compiled human PPI network data. The cancer disease ER genes and the developmental disease ER genes were found to be significantly more interconnected than would be expected by chance. Thus, rather than being randomly distributed within the human protein interaction network, ER-related disease genes form local connections to each other because they coherently contribute to disease risk through interactive co-function and co-regulation.

The CDEN and DDEN PPI subnetworks were identified by expanding the ER seed protein sets with the 100 iterations of the disease module detection (DIAMOnD) method [24]. Pathway enrichment analysis revealed that several signaling pathways related to epigenetic regulations were highly enriched in genes associated with both CDEN and DDEN. However, distinct pathways were also revealed between the CDEN and DDEN genes. The CDEN was significantly enriched for genes involved in SUMOylation, DNA repair, and cellular responses to stress. The SUMOylation and DNA repair pathways are essential for cell cycle progression and are highly deregulated within cancer tissues [53,54]. In contrast, the DDEN was significantly enriched for genes involved in HIV transcription initiation, RNA polymerase II HIV promoter escape, and RNA polymerase II transcription initiation. These pathways are mostly implicated in the transcriptional regulation of genes. Since dysregulation of gene transcription is a known cause of many developmental diseases [55], DDEN pathways play major roles in transcriptional abnormalities underlying the disorders. Collectively, these results suggest that the CDEN and the DDEN genes have shared molecular pathways in epigenetic regulations but are also associated with distinct pathways, implicating these subnetworks in the development of different diseases. The member genes of the distinct pathways might play critical roles that differentially induce cancer or developmental disease, and this potential warrants further investigation.

The analysis of functional relationships by means of a gene co-expression network has already been proven to be a useful tool for analyzing various disease-related gene functions [28,56]. While it was previously shown that a subset of ER genes were coexpressed as modules within multiple tissues [14], it was not determined whether the ER-associated co-expression modules exist at various stages of development. In the present study, WGCNA analysis was performed to identify gene co-expression modules from the published mouse developmental stage gene expression data. We identified co-expression modules containing the CDEN and DDEN genes in the early developmental stage of mouse embryos. The brown module was the most significant module identified for CDEN and was mainly enriched for genes involved in epigenetic gene regulatory processes such as histone modification and covalent chromatin modification. The expression profile of the brown module eigengene showed higher expressions during early developmental stages before the blastocyst stage. In the early stages of development, only cell division proceeds without cellular differentiation, suggesting that the epigenetic gene regulatory processes in the brown module might be mainly related to cell cycle progression. By contrast, the turquoise module was the most significant module identified for DDEN and was enriched for genes involved in processes such as axon development, axonogenesis, pattern specification, regionalization, and morphogenesis of the epithelium. These biological processes are all related to cellular differentiation and morphogenesis at the multicellular level. Moreover, the expression of the turquoise module eigengene was increased after the blastocyst stage, which is the developmental stage at which multicellular differentiation and morphogenesis begin. These observations suggest that turquoise module genes possess highly diversified functions that regulate multicellular development, which may imply that DDEN genes contribute to disease risk through defects in transcriptional regulation during multicellular developmental stages [6,13].

Our evolutionary analysis revealed that a greater level of purifying selection was acting upon the CDEN and DDEN gene sets, which had lower dN/dS ratios compared with those of the background and ER genes. Additionally, the negative Tajima’s *D*-values probably indicate that the CDEN and DDEN genes are still under ongoing purifying selection in the human population. Consistent with the evolutionary conservation, the evolutionary ages of the CDEN and DDEN genes were significantly older than that of the background genes. In general, these results for the CDEN and DDEN gene sets agree with the previous observations regarding epigenetic regulation genes in posttraumatic stress disorder [57]. Another previous study demonstrated that the evolutionary origins of heritable genetic disease genes tend to be ancient, originating with the early metazoans [58]. The present analysis indicated that the origins of the CDEN, DDEN, and ER genes are all more ancient still: these genes likely originated in unicellular eukaryotes. Thus, it was suggested that ER-related disease genes such as those in the CDEN and DDEN gene sets may constitute a significantly older group of disease genes.

Although the main selective force in ERs is purifying selection as estimated from sequence divergence (dN/dS), the present results also indicate that genes encoding ERs associated with disease are more likely to show evidence of recent positive selection inferred from polymorphism data. When analyzing primate divergence data, we observe the whole process of adaptation acting on protein-coding nucleotide sequences that facilitate the species differentiation, whereas recent human-specific positive selection inferred from polymorphism data reflects the recent adaptation of human populations to a wide range of new environments [59]. This difference in selection scenario might suggest a role for positively selected ER genes in achieving human fitness to the local environments. Previous investigations indicated that events of recent positive selection in the human populations mainly targeted cis-regulatory regions of the genes [60,61]; thus positive selection might efficiently tune the expression level of ER genes against various local environments. On the other hand, genes that control more than one trait may be prone to antagonistic pleiotropy, where at least the regulation of one trait has a positive effect on fitness, but also has a negative impact on other traits [62,63]. Because ERs putatively control the expression of many genes, it is very likely that positively selected ER genes are prone to antagonistic pleiotropy. We found the most significant enrichment of the gene set of positive selection in Seed-DDEN, which included 100 ERs highly shared among many developmental diseases. Moreover, Seed-DDEN genes were overrepresented in the turquoise co-expression module. Of the 55 Seed-DDEN genes in the turquoise module, 7 ER genes (*RAI1*, *KAT6B*, *NSD2*, *BAZ1B*, *SRCAP*, *ADNP*, and *HUWE1*) were found to be positively selected (Table 1). Most of them were implicated in the transcriptional regulation of multicellular development including DNA damage responses [64,65] and neural differentiation [66,67]. Therefore, while these ER genes appear to confer fitness advantages to humans during some stages of life history, they could also specifically increase the risk of various developmental diseases through the dysregulation of the turquoise module at the stages of multicellular development [68,69]. It would be quite interesting to characterize the regulatory regions of these positively selected ER genes and compare them with those of closely related primates in order to understand the consequences of positive selection on both human fitness and diseases.

In summary, we carried out a system-level analysis of epigenetic regulator genes and detailed the PPI subnetwork characteristics of ERs. We found that diseases associated with ERs are mainly clustered in two large groups, cancer diseases and developmental diseases. Our results showed that ER genes formed a highly interconnected PPI subnetwork, indicating a high tendency to interact and agglomerate with one another. We used the DIAMOnD algorithm to expand the PPI subnetworks into comprehensive ER-related disease gene networks, CDEN and DDEN. GO annotation of the gene co-expression modules associated with CDEN and DDEN genes revealed features related to the potential role of ER-related disease genes during early embryonic development. Although the results derived from the evolutionary rate and those from the evolutionary age analysis indicated the stronger evolutionary conservation of ER-related disease genes, we also observed statistically significant enrichment of the gene set of positive selection from polymorphism data in DDEN and Seed-DDEN. These findings shed light on the molecular mechanisms underlying the functions of ERs in various diseases and may help to identify new epigenetic disease-risk genes.

## Figures and Tables

**Table 1:** Genes that show evidence of positive selection in Seed-DDEN by polymorphism data.

Symbol	Gene Name	Functional Category	Turquoise Module
*ADNP*	activity dependent neuroprotector homeobox	Chromatin remodeling	+
*ARID1A*	AT-rich interaction domain 1A	Chromatin remodeling	−
*ARID1B*	AT-rich interaction domain 1B	Histone modification	−
*BAZ1B*	bromodomain adjacent to zinc finger domain 1B	Histone modification	+
*CREBBP*	CREB binding protein	Histone modification	−
*HUWE1*	HECT, UBA and WWE domain containing E3 ubiquitin protein ligase 1	Histone modification	+
*KANSL1*	KAT8 regulatory NSL complex subunit 1	Histone modification	−
*KAT6B*	lysine acetyltransferase 6B	Histone modification	+
*NIPBL*	NIPBL cohesin loading factor	Histone modification	−
*NSD2*	nuclear receptor binding SET domain protein 2	Histone modification	+
*RAI1*	retinoic acid induced 1	Chromatin remodeling	+
*SMARCA2*	SWI/SNF related, matrix associated, actin dependent regulator of chromatin	Histone modification	−
*SRCAP*	Snf2 related CREBBP activator protein	Chromatin remodeling	+
*VRK1*	VRK serine/threonine kinase 1	Histone modification	−
*ZMYND11*	zinc finger MYND-type containing 11	Histone modification	−

## Supplementary Materials

The following are available online at https://www.mdpi.com/2073-4425/11/12/1457/s1: Figure S1: Gene numbers and pathway annotation of the cancer disease seed genes and developmental disease seed genes. Figure S2: Expression profiles of the CDEN and DDEN genes across 32 human tissues. Figure S3: The WGCNA of the transcriptome in the early mouse developmental stages identified 21 co-expression modules. Figure S4: Incomplete HB scores for five sets of ER-related genes and background genes. Table S1: Epigenetic regulators (ERs) used in the present study. Table S2: Diseases enriched with ER genes. Table S3: Constituents of the cancer disease cluster and developmental disease cluster. Table S4: The 100 genes that were the most highly shared among diseases in the cancer disease cluster and the 100 that were most highly shared among diseases in the developmental disease cluster. Table S5: Reactome pathways for the CDEN and DDEN gene sets. Table S6: The human PPI network data. Table S7a: The cancer disease ER network (CDEN) data. Table S7b: The developmental disease ER network (DDEN) data. Table S8: The 21 co-expression modules identified using WGCNA.
