# Supplementary material for: Network and Evolutionary Analysis of Human Epigenetic Regulators to Unravel Disease Associations
Source: Genes (Basel). 2020 Dec 4;11(12):1457. doi: 10.3390/genes11121457 (PMC7761991; doi:10.3390/genes11121457)

A

Module	No. of Genes	Module	No. of Genes	Module	No. of Genes
black	371	grey60	73	purple	184
blue	3102	lightcyan	81	red	732
brown	1417	lightgreen	69	royalblue	60
cyan	108	lightyellow	65	salmon	109
green	1209	magenta	225	tan	117
greenyellow	161	midnightblue	105	turquoise	8595
grey	797	pink	249	yellow	1358

B

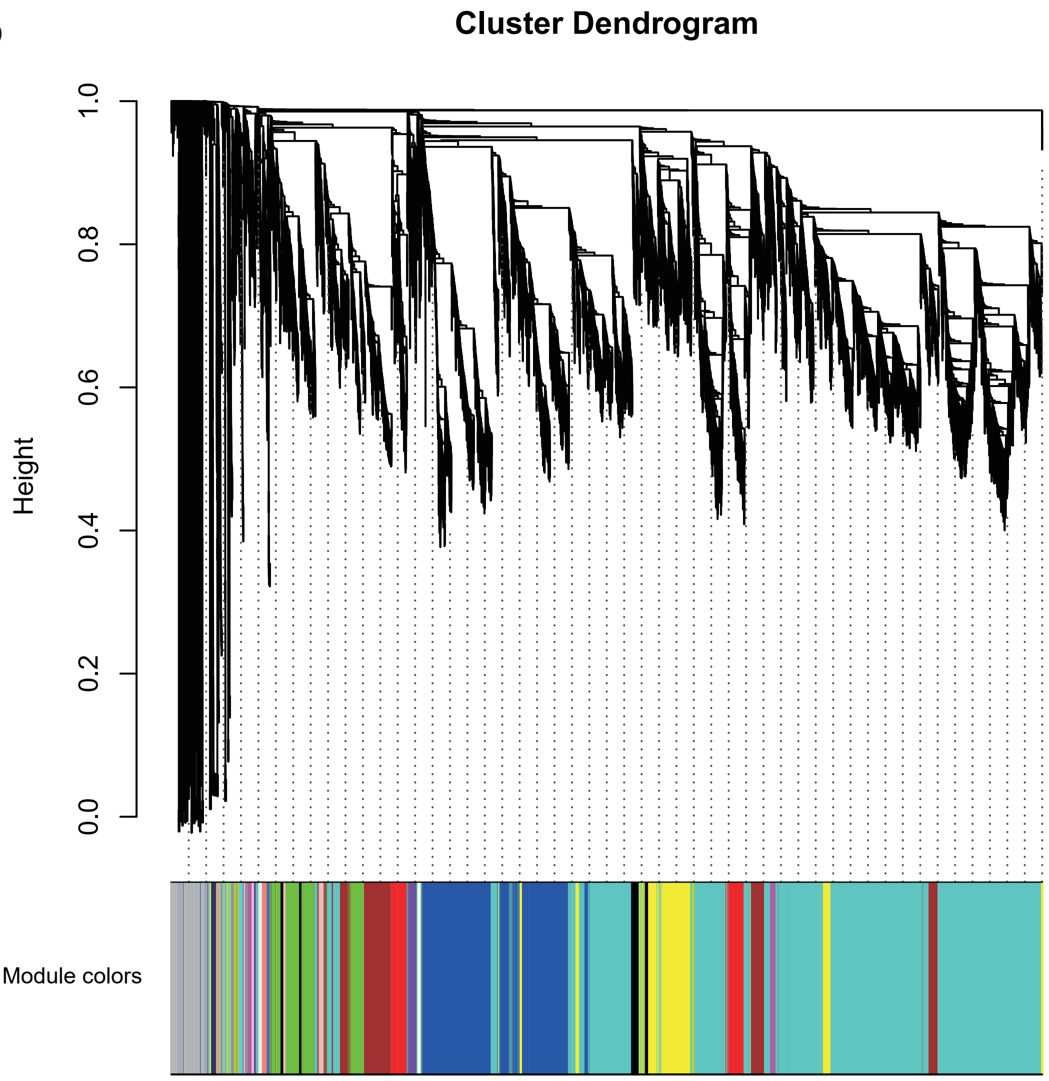

Supplement: Supplementary file 1 [file genes-11-01457-s001.zip › Supplementary files/Figure_S3.pdf]
