# Supplementary material for: Network and Evolutionary Analysis of Human Epigenetic Regulators to Unravel Disease Associations
Source: Genes (Basel). 2020 Dec 4;11(12):1457. doi: 10.3390/genes11121457 (PMC7761991; doi:10.3390/genes11121457)
Supplement: Supplementary file 1 [file genes-11-01457-s001.zip › Supplementary files/Legends for Supplementary files.docx]

**Legends for Supplementary files**

**Figure S1**

Gene numbers and pathway annotation of the cancer disease seed genes and developmental disease seed genes. (A) The number of overlapping genes between the cancer disease seed genes and developmental disease seed genes. (B) Pathway analysis of the cancer disease seed and developmental disease seed data sets. Reactome pathway terms were determined for each gene set using the compareCluster function in the R package clusterProfiler. The most overrepresented Reactome terms are illustrated as dot plots, with the gene ratio denoted by size and significance denoted by color. *p*‑values were adjusted by the Benjamini‑Hochberg method.

**Figure S2**

Expression profiles of the CDEN and DDEN genes across 32 human tissues. (A) Gene expression levels of ER, CDEN, DDEN and background genes across 32 human tissues. For each tissue, the median expression of the genes in a gene set was calculated to represent the expression level. (B) Composition of gene categories with different tissue specificity in the ER, CDEN, DDEN and background gene sets.

**Figure S3**

The WGCNA of the transcriptome in the early mouse developmental stages identified 21 co-expression modules. (A) The number of member genes in each module represented by color. (B) Clustering dendrogram of the genes showing module membership by color.

**Figure S4**

Incomplete HB scores for five sets of ER-related genes and background genes. We show the HB scores for the three populations of the 1000 Genomes Project Phase 1: Utah residents with Northern and Western European Ancestry (CEU), Han Chinese in Beijing, China (CHB), and Yoruba from Ibadan, Nigeria (YRI). An asterisk indicates *p* < 0.05 compared with background genes (Mann-Whitney U test).

**Table S1**

Epigenetic regulators (ERs) used in the present study.

**Table S2**

Diseases enriched with ER genes. A total of 457 diseases at *q* values greater than 0.05 are shown with the associated ER genes.

**Table S3**

Constituents of the cancer disease cluster and developmental disease cluster. Diseases in the cancer disease cluster and developmental disease cluster are shown with the associated ER genes.

**Table S4**

The 100 genes that were the most highly shared among diseases in the cancer disease cluster and the 100 that were most highly shared among diseases in the developmental disease cluster.

**Table S5**

Reactome pathways for the CDEN and DDEN gene sets. Pathways at adjusted *p*-values greater than 0.05 are shown with associated genes.

**Table S6**

The human PPI network data. Each line in the table correponds to one interaction. Columns 1 and 2 give the gene IDs of the two interacting proteins.

**Table S7a**

The cancer disease ER network (CDEN) data. Each line in the table correponds to one interaction. Columns 1 and 2 give the gene IDs of the two interacting proteins.

**Table S7b**

The developmental disease ER network (DDEN) data. Each line in the table correponds to one interaction. Columns 1 and 2 give the gene IDs of the two interacting proteins.

**Table S8**

The 21 co-expression modules identified using WGCNA.
