# Supplementary figures and images for: Network and Evolutionary Analysis of Human Epigenetic Regulators to Unravel Disease Associations
Source: Genes (Basel). 2020 Dec 4;11(12):1457. doi: 10.3390/genes11121457 (PMC7761991; doi:10.3390/genes11121457)

# A

Cancer disease genes

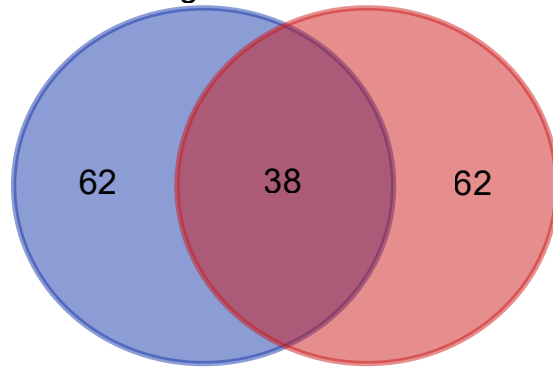

developmental disease genes

# B

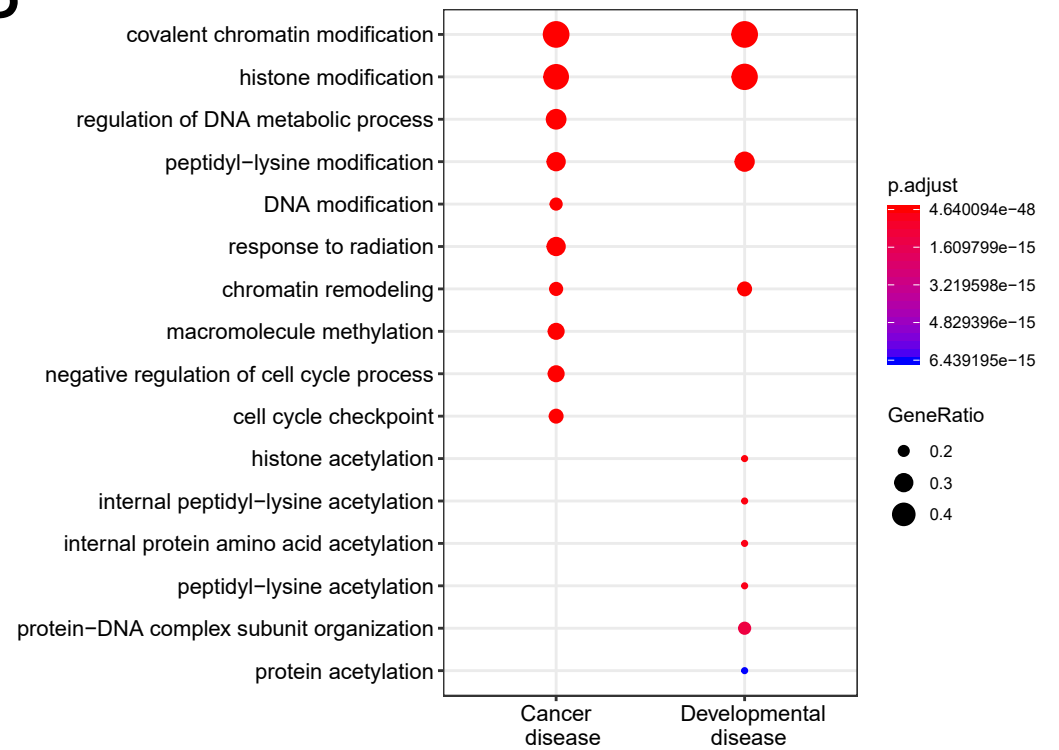

Supplement: Supplementary file 1 [file genes-11-01457-s001.zip › Supplementary files/Figure_S1.pdf]

A

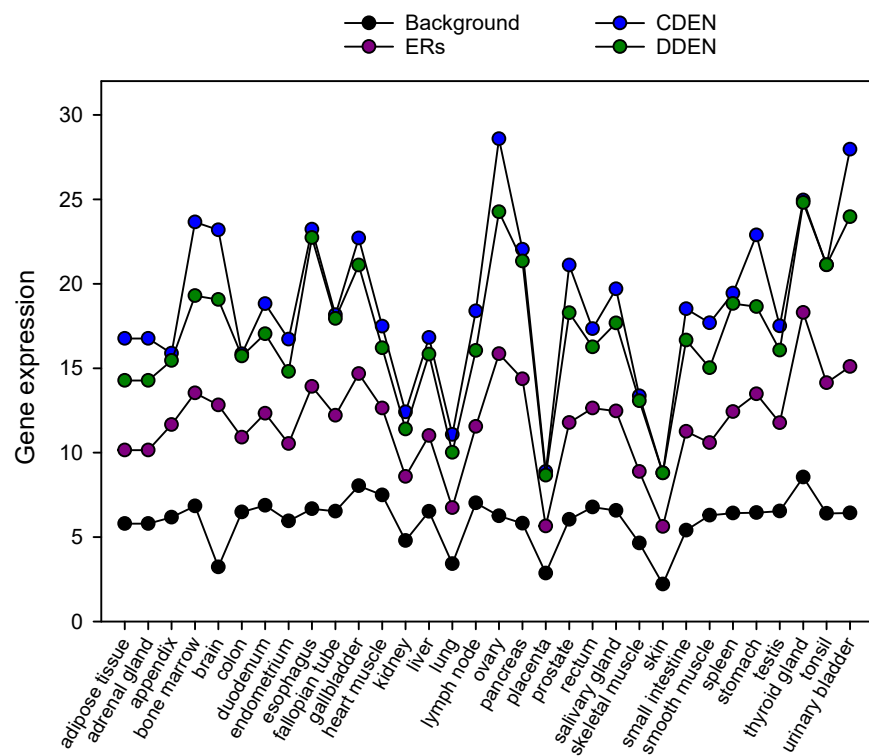

B

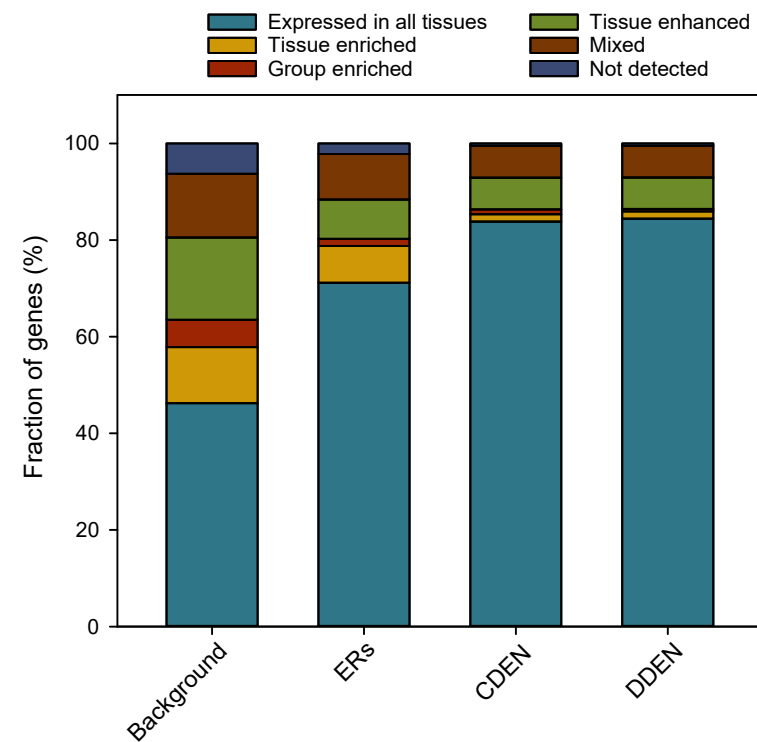

Supplement: Supplementary file 1 [file genes-11-01457-s001.zip › Supplementary files/Figure_S2.pdf]

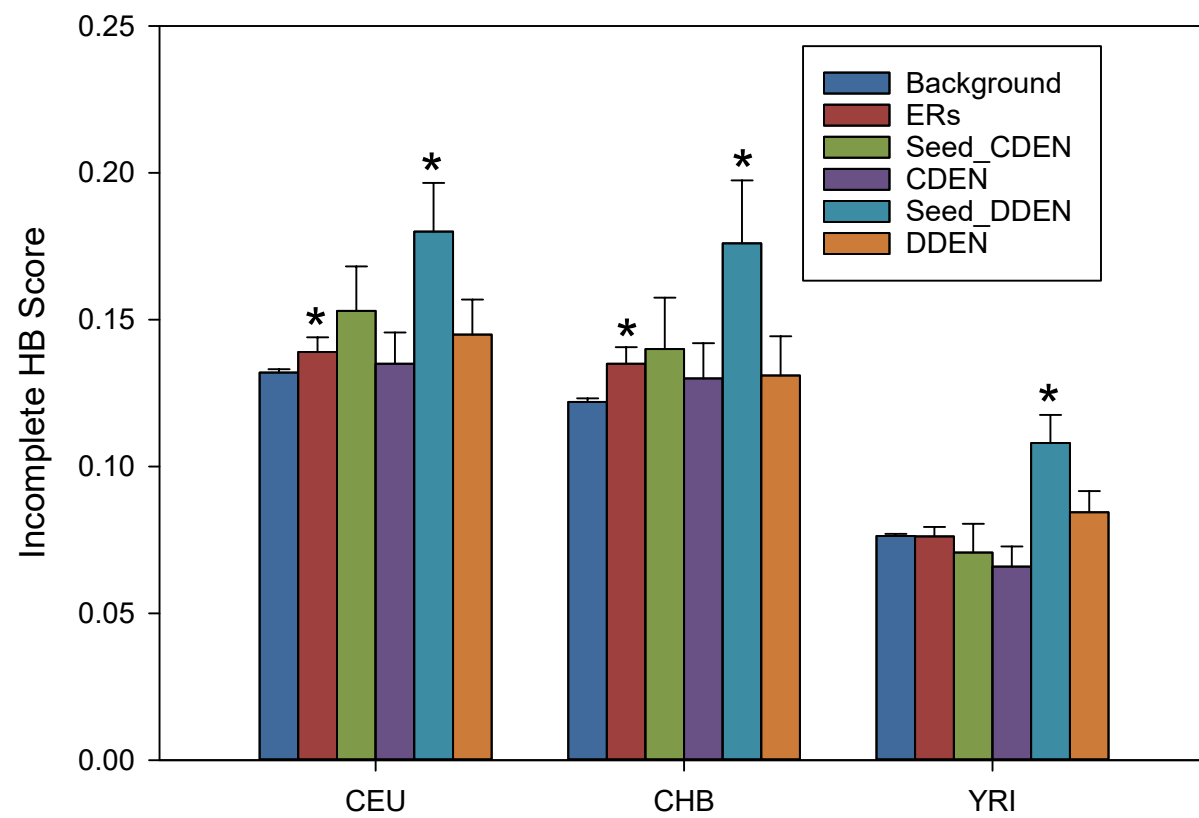

Supplement: Supplementary file 1 [file genes-11-01457-s001.zip › Supplementary files/Figure_S4.pdf]
